# Two cases of esophageal basaloid squamous cell carcinoma which achieved long‐term survival by endoscopic submucosal dissection and additional chemoradiotherapy

**DOI:** 10.1002/deo2.211

**Published:** 2023-01-31

**Authors:** Yoshiki Morihisa, Hiroyoshi Iwagami, Takuji Akamatsu, Shogo Nakano, Midori Wakita, Takeya Edagawa, Takafumi Konishi, Yasuki Nakatani, Yukitaka Yamashita

**Affiliations:** ^1^ Department of Gastroenterology and Hepatology Japanese Red Cross Wakayama Medical Center Wakayama Japan

**Keywords:** basaloid squamous cell carcinoma, chemoradiotherapy, endoscopic submucosal dissection, esophagogastroduodenoscopy, submucosal tumor

## Abstract

Herein, we report two rare basaloid squamous cell carcinoma (BSCC) cases. Esophagogastroduodenoscopy revealed a submucosal tumor‐like lesion and a biopsied specimen showed a finding suspected of BSCC in both cases. Both lesions underwent endoscopic submucosal dissection with en bloc resection, and long‐term survival was achieved using additional chemoradiotherapy. The standard treatment for BSCC has not been determined, and there are few reports of esophageal BSCC treated using endoscopic resection. Endoscopic submucosal dissection and additional chemoradiotherapy for superficial BSCC may be effective treatment options.

## INTRODUCTION

Esophageal basaloid squamous cell carcinoma (BSCC) is an extremely rare histological variant of squamous cell carcinoma (SCC), accounting for 0.068%–4% of all malignant esophageal tumors.^1^ BSCC occurs in the lamina propria mucosae, is covered with non‐cancerous epithelium, and grows like a submucosal tumor (SMT). Because BSCC has such growth and macroscopic features, it is difficult to detect it early; therefore, it is generally detected at an advanced stage. However, because the prognosis is relatively good in cases of BSCC detected and treated early,^2^ it is important to recognize the endoscopic characteristics and strive for early detection. Herein, we report two rare BSCC cases that achieved long‐term survival after treatment using endoscopic submucosal dissection (ESD) and chemoradiotherapy (CRT).

## CASE REPORT

### Case 1

**FIGURE 1:**
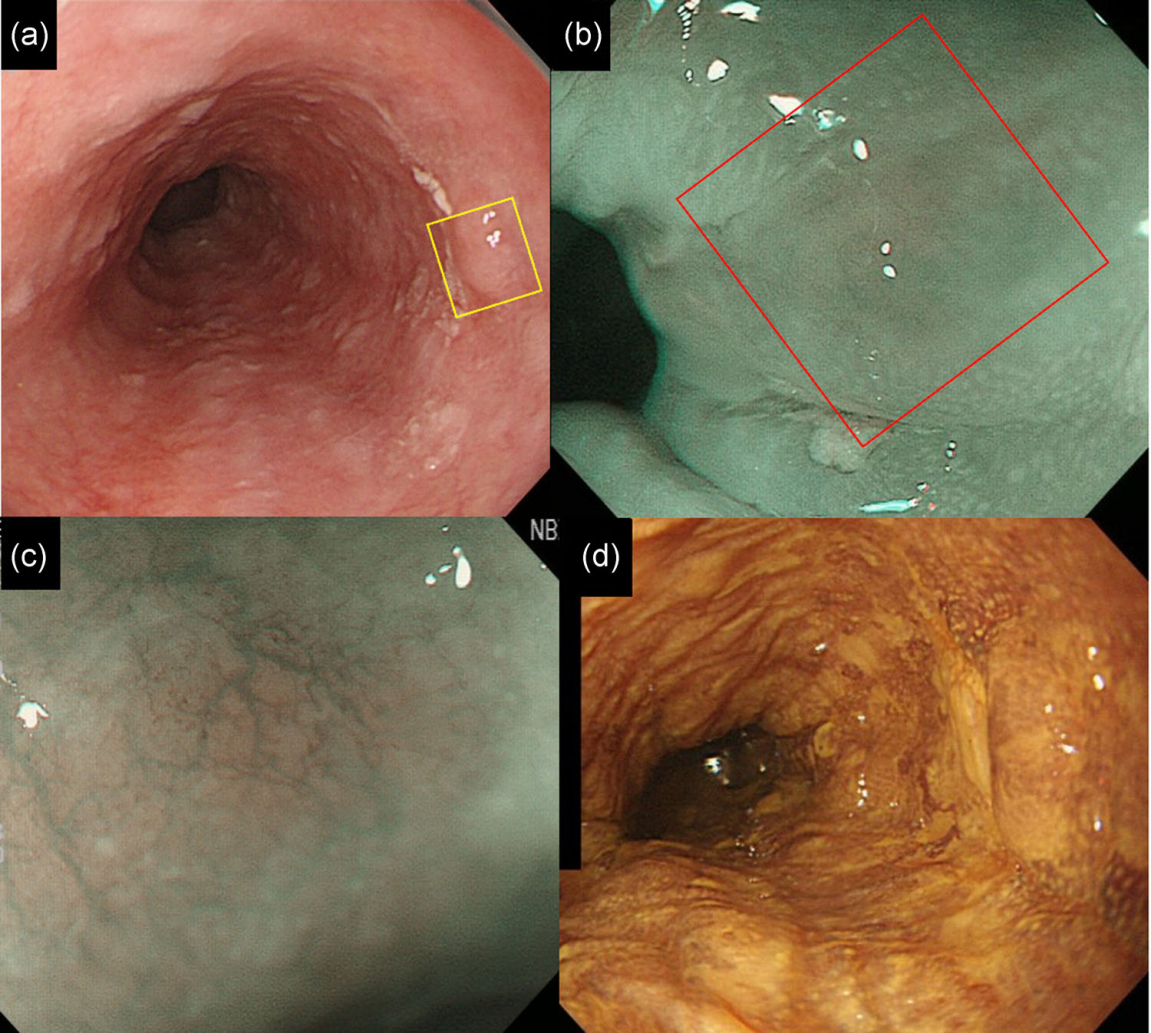
(a) White light imaging showing a submucosal tumor‐like protrusion in the lower thoracic esophagus. (b) Magnified image of the yellow square in Figure 1a. Narrow‐band imaging showing a slight brownish area. (c) Magnified image of the red square in Figure 1b. Narrow‐band imaging showing Type A of intrapapillary capillary loop according to the Japan Esophagus Society classification. (d) Lugol chromoendoscopy showing poor staining of the lesion and its surrounding area.

**FIGURE 2:**
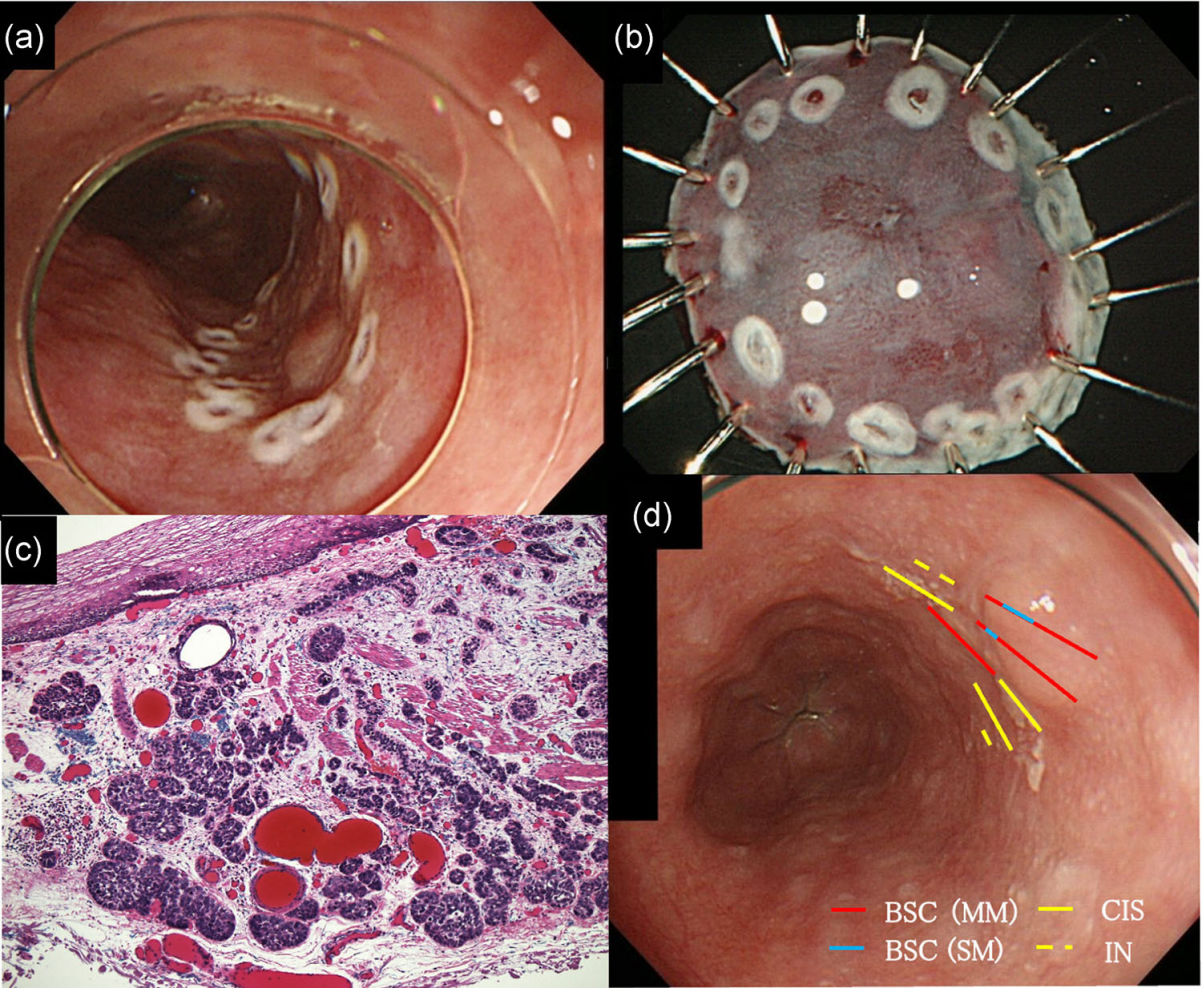
(a) We marked a slightly wide margin around the lesion, including the Lugol unstained area and the protruded part. (b) We achieved en bloc resection. (c) Magnified image of the lesion. Hematoxylin‐eosin staining (HE × 100) showing basal cell‐like hyperchromatic crowded tumor cells that proliferated mainly in the lamina propria. The tumor surface was covered with normal squamous epithelium. (d) Mapping of endoscopic images based on histopathological examination. Basaloid squamous cell carcinoma is distributed in the submucosal tumor‐like protruding part. The red and blue lines are the areas of the muscularis mucosae and submucosal layer of the basaloid squamous cell carcinoma depth, respectively. The yellow line represents the area of carcinoma in situ or intraepithelial neoplasia around the BSCC area.

A 79‐year‐old man had a history of smoking (60 pack‐years) and social drinking. We detected a 5‐mm‐sized lesion in the lower thoracic esophagus, which was SMT‐like and protruding, using white light imaging of esophagogastroduodenoscopy (Figure 1a). Narrow‐band imaging revealed that the lesion had a slightly brownish area. The intrapapillary capillary loop found around the lesion was type A according to the Japan Esophagus Society classification^3^ (Figure 1b,c). According to Lugol chromoendoscopy, the lesion and its surrounding area showed poor staining (Figure 1d). According to endoscopic ultrasonography, although the submucosal layer was compressed by the low echoic mass, it was not broken (Figure S1); therefore, we considered that it was possible to resect the lesion safely using ESD. The biopsied specimen showed poorly differentiated tumor cells (Figure S2). We suspected BSCC as the differential diagnosis and considered the clinical stage to be T1aN0M0. ESD was performed for accurate diagnosis and to determine the tumor's invasion depth. We marked a slightly wide margin around the lesion, including the Lugol poorly stained area, and the protruded part and achieved en bloc resection (Figure 2a,b). Histological examination of the resected specimen revealed basal cell‐like hyperchromatic tumor cells that proliferated mainly in the lamina propria, and the tumor's surface was covered with non‐neoplastic lesions (Figure 2c). Figure 2d shows the mapping of the tumor on the endoscopic image. Carcinoma in situ or intraepithelial neoplasia was present in the area around the protruding SMT‐like part. The final pathological diagnosis was BSCC: 0‐I+IIb, 7 mm, pT1b (SM2 0.4 mm), ly0, v0, INF b, pHM0, pVM0, and pStage I. Two months after ESD, the patient was treated using concurrent CRT. The CRT regimen comprised cisplatin (70 mg/m^2^) on days 1 and 29, 5‐fluorouracil (700 mg/m^2^) on days 1–4 and 29–32, and 60 Gy radiotherapy (RT) in 30 fractions. No recurrence was observed during the 7‐year follow‐up.

### Case 2

**FIGURE 3:**
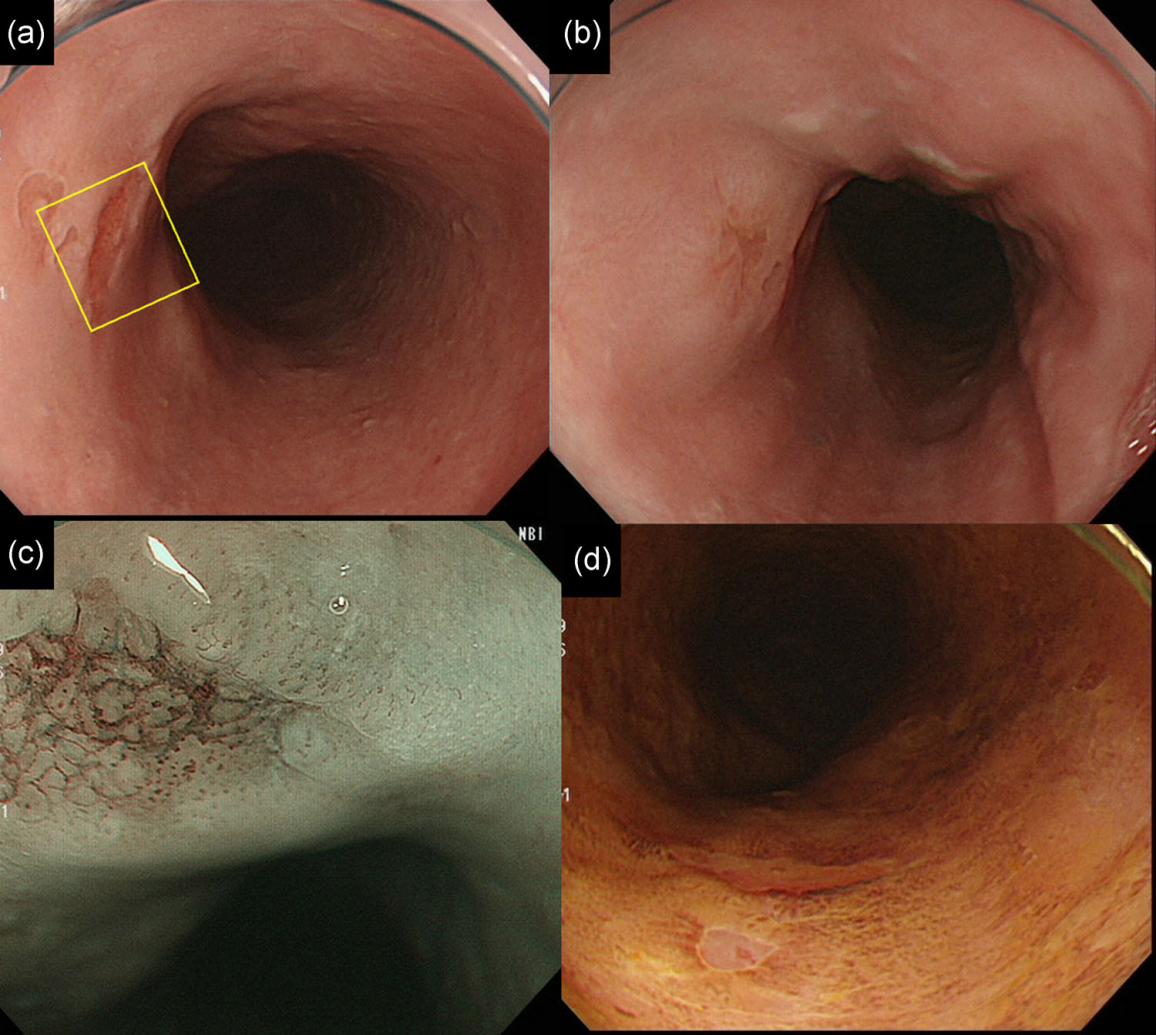
(a, b) White light imaging showing a submucosal tumor‐like protrusion with depression in the upper thoracic esophagus. (c) Magnified image of the yellow square in Figure 3a. Narrow‐band imaging showed a brownish area at the depressed part and Type B1 with a small avascular area according to the Japan Esophagus Society classification. (d) Lugol chromoendoscopy showing the depression area was unstainied.

**FIGURE 4:**
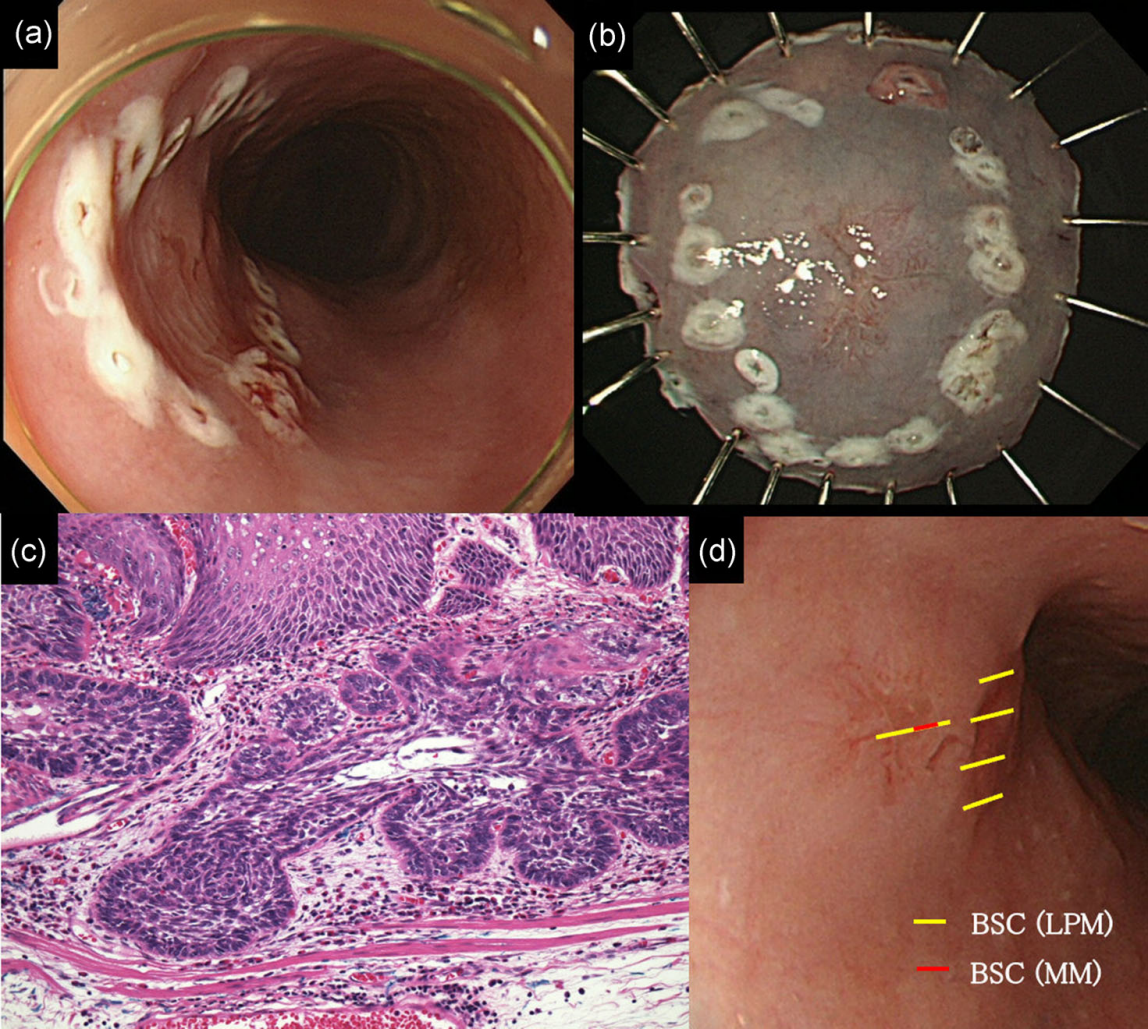
(a) We marked a slightly wide margin around the lesion, including the protruded part. (b) We achieved en bloc resection. (c) Magnified image of the lesion. Hematoxylin‐eosin staining (HE × 200) showing that tumor cells resembling basal cells formed solid nests and lobule structures in a ribbon‐like arrangement. (d) Mapping of endoscopic images based on histopathological examination. The basaloid squamous cell carcinoma (BSCC) was distributed at the submucosal tumor (SMT)‐like protruded part and depressed parts. The yellow and red lines indicate the areas of the lamina propria mucosa and muscularis mucosae of basaloid squamous cell carcinoma depth, respectively.

A 66‐year‐old man had a history of smoking (44 pack‐years) and daily drinking. We detected a 5‐mm‐sized lesion in the upper thoracic esophagus, which was SMT‐like and protruding with depression at the center, using white light imaging (Figure 3a,b). Narrow‐band imaging revealed that the intrapapillary capillary loop was type B1 with a small avascular area^3^ (Figure 3c). According to Lugol chromoendoscopy, the depression at the center of the lesion was unstained (Figure 3d). Endoscopic ultrasonography showed no remarkable finding suspected of submucosal invasion (Figure S3). The biopsied specimen showed irregular proliferation of atypical cells with lobule structures (Figure S4). We suspected the lesion was BSCC and considered the clinical stage to be T1aN0M0. We marked a slightly wide margin around the lesion like in Case 1, and ESD was performed (Figure 4a). We achieved en bloc resection (Figure 4b). Histological examination of the resected specimen showed that tumor cells resembling basal cells formed solid nests and lobule structures in a ribbon‐like arrangement. The lobules of the tumor cells displayed peripheral nuclear palisading (Figure 4c). Figure 4d shows the mapping of the tumor on the endoscopic image. The BSCC existed in the normal epithelium around the depression. The final pathological diagnosis was BSCC: 0‐IIc, 8 mm, pT1a (MM), ly0, v0, INF a, pHM0, pVM0, and pStage 0. Two months after the ESD, the patient was treated using concurrent CRT. The regimen comprised cisplatin (70 mg/m^2^) on days 1 and 29, 5‐fluorouracil (700 mg/m^2^) on days 1–4 and 29–32, and 41.4 Gy RT in 23 fractions. No recurrence was observed during the 7‐year follow‐up.

## DISCUSSION

We experienced two superficial esophageal BSCC cases that achieved long‐term survival after treatment using ESD and additional CRT.

Esophageal BSCC, a rare variant of SCC, is most common in the middle thoracic esophagus.^1^ Smoking, alcohol, and aging (especially in males) are major risk factors for BSCC, similar to typical SCC. Macroscopic findings of superficial esophageal BSCC revealed it is mainly SMT‐like protruding and covered with normal epithelium.^2^ Furthermore, some cases have erosion or depression at the center of the protruded part.^4^ While no characteristic findings exist on magnified narrow‐band imaging, some cases have brownish areas that indicate intraepithelial neoplasia around the SMT‐like protruding, as in Case 1. In addition, no Lugol staining is often observed in BSCC, similar to typical SCC.

It is difficult to diagnose BSCC accurately using endoscopically biopsied specimens. This is because BSCCs are derived from basal cells in the deepest area of the epithelial layer, and only superficial and small parts of the tumor can be obtained using endoscopic biopsy. In addition, the diagnostic accuracy of BSCC using biopsied specimens is reported to be only 4.7%–10%.^5^ However, we could suspect BSCC using a biopsied specimen. We speculated that this is because we could take biopsies in a sufficient amount from the Lugol unstained area in Case 1, whereas in Case 2, biopsies were taken from the depressed part where BSCC was exposed on the surfaces. Therefore, it is important to take biopsies in a sufficient amount from erosion or Lugol unstained areas to diagnose BSCC. However, when a definitive diagnosis is difficult, ESD is one of the options for both accurate diagnosis and treatment.

Generally, BSCC is reported to have a poor prognosis, high proliferative activity, and a high incidence of lymph nodes and distant metastases.^2^ However, early‐stage BSCC is associated with lymph node metastasis, and its prognosis is as low as that of typical SCC. In a review of 60 esophageal BSCCs reported by Yoshioka et al.,^4^ the survival rates of patients with early‐stage BSCC (stage I or II) were similar to those of patients with typical SCC. Therefore, because early diagnosis of BSCC is important to improve prognosis, recognizing the above risk factors and endoscopic findings is required.

There is no consensus on the standard treatment for esophageal BSCC^1^ because it is usually detected at an advanced stage, and surgical resection or CRT is generally performed because there are some reports that CRT and neoadjuvant chemotherapy are effective.^6^, ^7^ However, to the best of our knowledge, only seven cases of BSCC treated using endoscopic resection (ER) have been reported in the English literature, including our cases.^8^ Among the seven cases reviewed, endoscopic mucosal resection was performed in two cases, and ESD was performed in the remaining five (Table S1). Based on the pathological findings, the invasion depth in all cases was limited to MM to SM2. The horizontal and vertical margins were negative in all the cases. However, it is often difficult to accurately diagnose the lateral extent of the BSCC based on endoscopic findings because the BSCC spreads laterally under the normal epithelium around the depression part, as described in Case 2. Therefore, it is necessary to maintain a wide resection margin from the tumor when ESD is attempted. As additional therapy, one patient underwent RT, and both patients in our report received CRT. We determined the radiation doses according to the local and distant metastasis recurrence risks. While the invasion depth in Cases 1 and 2 was SM2 and MM, respectively the risk of recurrence was reported to be 25.7% and 4.29%, respectively.^9^ However, Case 1 seemed to have a higher recurrence risk than Case 2. Finally, after consultation with the radiologist, we decided on the radiation doses. Three cases demonstrated no recurrence 6–35 months after ER.^8^ Regarding two cases in this report, no recurrence was found 7 years after ESD and additional CRT. In superficial BSCC cases, ESD and additional RT or CRT may contribute to long‐term survival.

In conclusion, when SMT‐like protrusion is observed, especially in the brownish area, which is suspected to be intraepithelial neoplasia around the protrusion, BSCC should be considered a differential diagnosis. A wide resection margin is important when performing ESD. ESD and additional CRT might be treatment options for superficial BSCC.

## CONFLICT OF INTEREST

None.

## Supporting information

Figure S1

Figure S2

Figure S3

Figure S4

Table S1
