# Supplementary figures and images for: Two cases of esophageal basaloid squamous cell carcinoma which achieved long‐term survival by endoscopic submucosal dissection and additional chemoradiotherapy
Source: DEN Open. 2023 Jan 31;3(1):e211. doi: 10.1002/deo2.211 (PMC9889968; doi:10.1002/deo2.211)

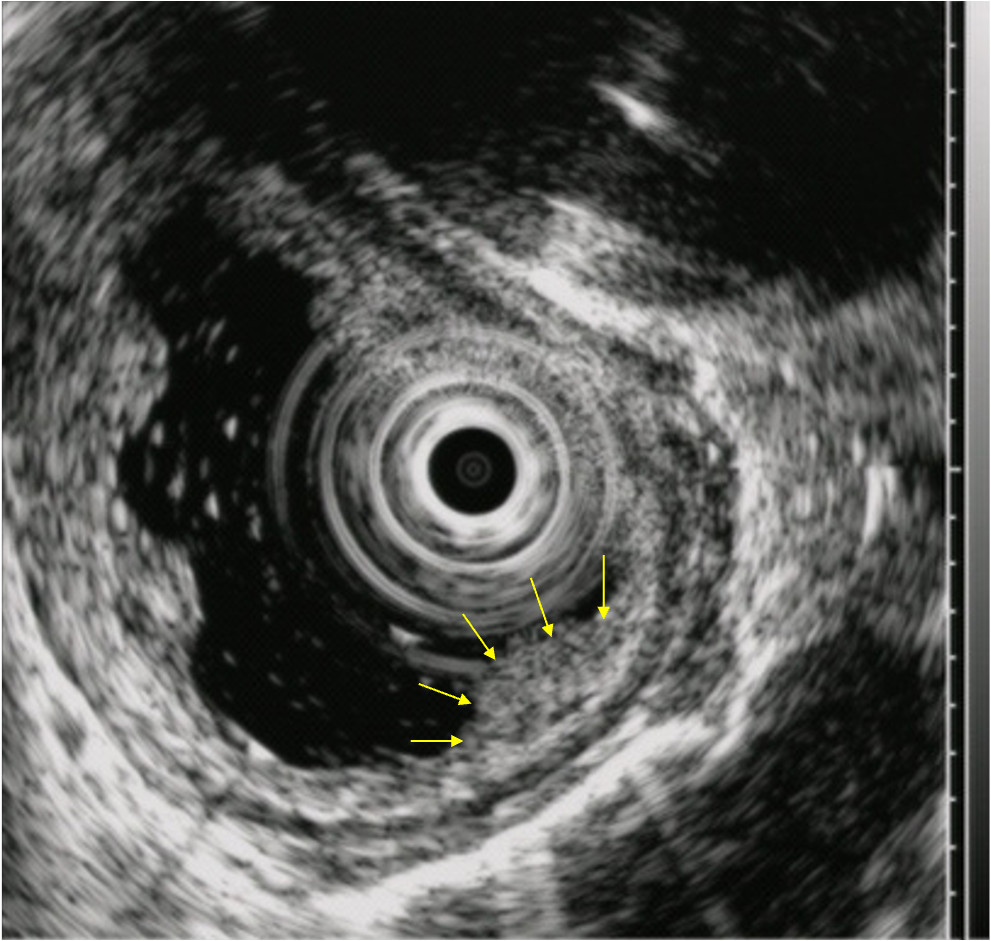

Supplement: Supplementary file 1 — Figure S1 [file DEO2-3-e211-s001.jpg]

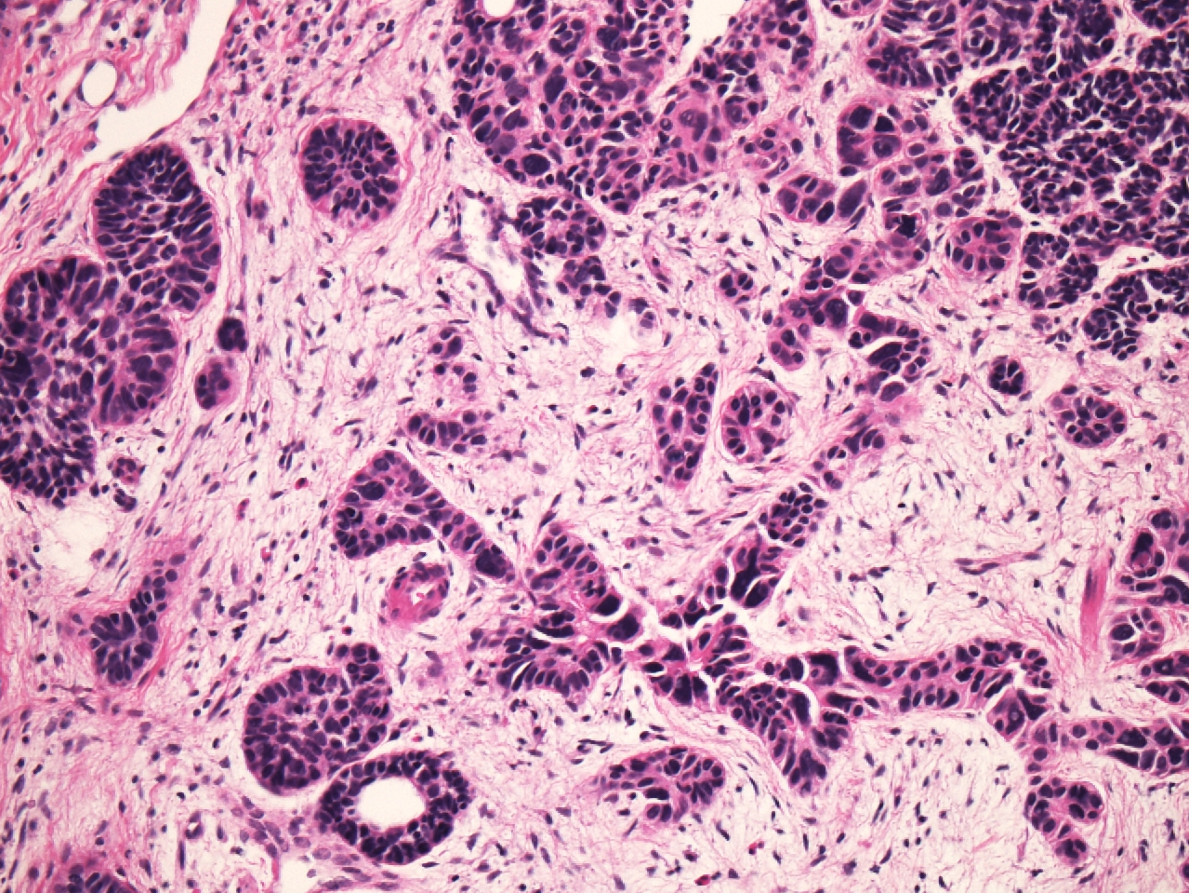

Supplement: Supplementary file 2 — Figure S2 [file DEO2-3-e211-s005.jpg]

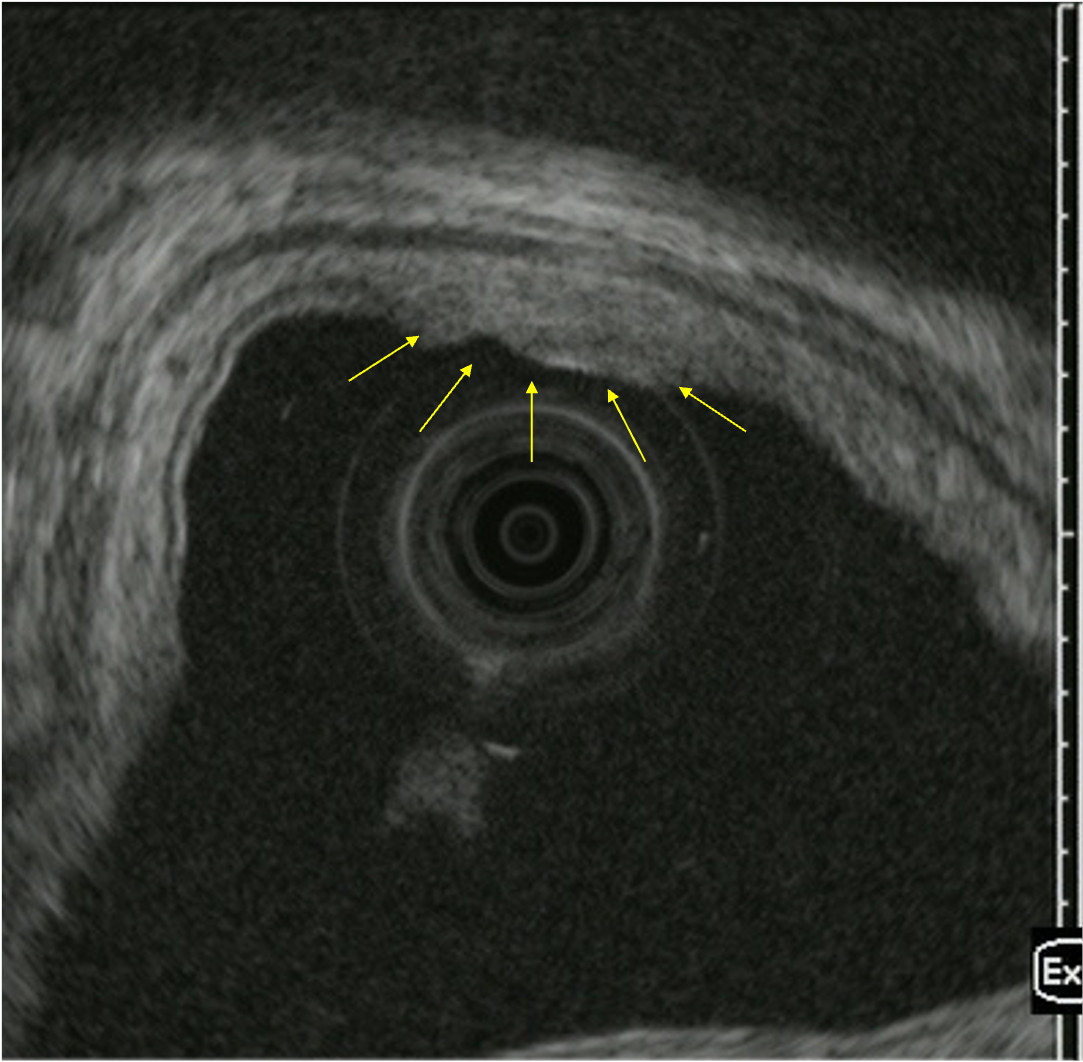

Supplement: Supplementary file 3 — Figure S3 [file DEO2-3-e211-s004.jpg]

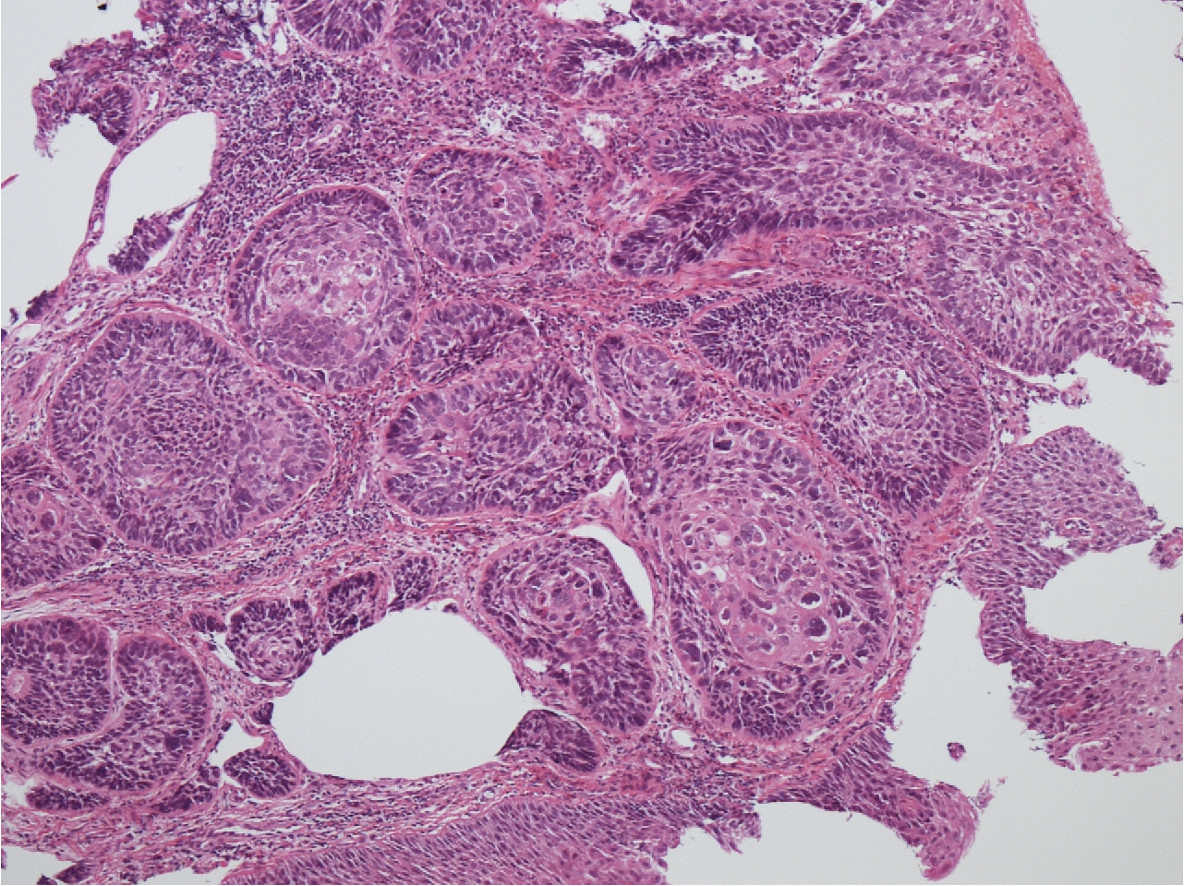

Supplement: Supplementary file 4 — Figure S4 [file DEO2-3-e211-s003.jpg]
