# Supplementary material for: Two cases of esophageal basaloid squamous cell carcinoma which achieved long‐term survival by endoscopic submucosal dissection and additional chemoradiotherapy
Source: DEN Open. 2023 Jan 31;3(1):e211. doi: 10.1002/deo2.211 (PMC9889968; doi:10.1002/deo2.211)
Supplement: Supplementary file 5 — Table S1 [file DEO2-3-e211-s002.docx]

**Supplementary Table 1**

Reported cases of basaloid squamous cell carcinoma that were resected endoscopically

| Author | Year | Age (years) | Sex | Location | Macroscopic type | Tumor size (mm) | Specimen size  (mm) | NBI-ME | Treatment | pT | Lymphovascular invasion | pStage | Follow up period | prognosis |
| --- | --- | --- | --- | --- | --- | --- | --- | --- | --- | --- | --- | --- | --- | --- |
| Nakamura et al | 2012 | 79 | M | Cervical | Isp | 7 | 20 | SECN | EMR | 1a-LPM | Negative | I | 6 months | No  recurrence |
| Kai et al | 2014 | 70 | M | Middle | Is | 5 | No data | B1 | ESD | 1b-SM2 | No data | I | No data | No data |
| Kim et al | 2018 | 59 | M | Lower | Isp | 11 | No data | SECN | EMR＋RT | 1b-SM2 | Negative | I | 35 months | No  recurrence |
| Di et al | 2019 | 62 | M | Middle | IIa | 5 | 22 | B1 | ESD | 1a-LPM | Negative | 0 | No data | No data |
| Shiratori  et al | 2021 | 87 | M | Middle | Is | 6 | No data | B2 | ESD | 1a-MM | Negative | 0 | 8 months | No  recurrence |
| Case 1 | 2022 | 79 | M | Lower | Is＋IIb | 7 | 25 | SECN | ESD＋CRT | 1b-SM2 | Negative | I | 7 years | No  recurrence |
| Case 2 | 2022 | 66 | M | Middle | IIc | 8 | 34 | B1 | ESD＋CRT | 1a-MM | Negative | 0 | 7 years | No  recurrence |

*EMR*, endoscopic mucosal resection; *ESD*, endoscopic submucosal dissection; *NBI-ME*, narrow band image with magnifying endoscopy; *SECN*, subepithelial capillary network; *LPM*, lamina propria musosae; *MM*, musclaris mucosae; *SM*, submucosa
